# Data on Growth, survivability, water quality and hemato-biochemical indices of Nile Tilapia (*Oreochromis niloticus*) fry fed with selected marine microalgae

**DOI:** 10.1016/j.dib.2021.107422

**Published:** 2021-09-22

**Authors:** Kafia Islam Amira, Mohammad Redwanur Rahman, Suchandan Sikder, Helena Khatoon, Jinat Afruj, Mohammad Ekramul Haque, Tashrif Mahmud Minhaz

**Affiliations:** a Department of Aquaculture, Chattogram Veterinary and Animal Sciences University, Chattogram 4225, Bangladesh; b Department of Medicine and Surgery, Chattogram Veterinary and Animal Sciences University, Chattogram 4225, Bangladesh

**Keywords:** Tilapia, Microalgae, Growth, Survivability, Water quality, Hemato-biochemical indices

## Abstract

Data of this article describes growth, survival rate, water quality and hemato-biochemical indices of Nile Tilapia (*Oreochromis niloticus*) fry. To collect the data, the Nile Tilapia fry was reared in 30 L glass aquarium (18 fish/ tank) for 56-days under controlled environmental condition. Feed was prepared with 25 and 50% replacement of commercial fish meal with *Nannochloropsis* sp. and *Tetraselmis* sp*.* microalgae, while no replacement was made for control feed. Initial and final body weight of fish was recorded to find the data of growth rate; survival rate was calculated from the initial and final live individuals recorded during the experiment; physico-chemical parameters were analyzed to collect water quality data; hemato-biochemical indices were collected using hematology analyzer and photometry. The data on growth, survival rate and hemato-biochemical indices were statistically significant (p < 0.05). Therefore, these data might contribute to the selection of marine microalgae to improve the water quality during fish farming which could enhance the growth and survivability of fish. In addition, the data of hemato-biochemical indices represent that feeding selected marine microalgae might result in the production of healthy and disease-free fish.

## Specifications Table

**Table:** 

Subject	Food Science, Aquatic Science
More specific subject area	Fish growth, survivability, water quality and hemato-biochemical indices
Type of data	Table and chart
How data were acquired	Data were obtained by physical measurements, calculation of survival and specific growth rate from experiments. Physical parameters for water quality such as temperature, pH, and dissolved oxygen were measured everyday by Multimeter. Chemical parameters, such as, total ammonia nitrogen, nitrite nitrogen, and phosphate phosphorous were measured by chemical methods using spectrophotometer. Data on hemato-biochemical parameters were collected using hematology analyzer and photometric method. Statistical analysis was performed using IBM SPSS software (v.26).
Data format	Raw and analyzed
Parameters for data collection	Mass culture of selected microalgae was done using Conway culture medium. Biomass was harvested at stationary phase by centrifugation. Harvested biomass was dried and feed was prepared with different level of inclusion of selected microalgae mixed-with commercial feed. Total of 270 Nile tilapia fry (18 per replicates) was randomly distributed in different treatment with three replicates. Data of physical parameters such as temperature, salinity, pH, and dissolved oxygen in the culture tanks were collected daily. On the other hand, chemical parameters ammonia, nitrite and soluble reactive phosphorous data were collected once in a week. In case of growth, survivability and hemato-biochemical indices data were collected at the end of the experiment. Duration of the experiment was 56 days.
Description of data collection	For specific growth rate: initial and final body weight of fish fry was measured. For survivability: number of live fish was counted at the end of the experiment. For water quality: temperature, pH, dissolve oxygen, total ammonia nitrogen, nitrite nitrogen, and soluble reactive phosphorus. For hematology: red blood cell, hemoglobin, packed cell volume, white blood cell, lymphocytes and platelet. For serum biochemistry: total protein, albumin, globulin, A/G ratio, blood glucose, triglyceride, cholesterol, urea, and blood urea nitrogen.
Data source location	Microalgae Research Corner and Wet Laboratory, Department of Aquaculture, Faculty of Fisheries, Chattogram Veterinary and Animal Sciences University, Khulshi-4225, Chattogram, Bangladesh
Data accessibility	Data are available with this article and also at https://data.mendeley.com/datasets/hv5fg5r869/1

## Value of the Data

• The present data signify the idea of selection of microalgae as feed supplement to improve the water quality during fish farming which will enhance the growth and survivability of fish. Hemato-biochemical data might aid in diagnosing diseases and examining the degree of blood cell loss to assess health conditions and physiological improvements of fry with diet intake in effective and comprehensive indexes.
• Farmers and researchers can utilize these data in understanding the systemic interactions between the homeostasis and physiological modifications consequently from diet and water quality of the fish species so that standard reference values for this fish can be established. They can also improve their yield and to conduct further investigation on the effect of other microalgae on fish health, disease resistance as well as yield performance, respectively.
• Researchers can use these data to understand and improve the path of generating alternative way of fish culture for high production and quality improvement in fish farming. It can be directed to the investigation of the effect of other marine or freshwater microalgae on the improvement of water quality and consequently, the improvement of yield of commercially important species. Assessment of the effectiveness of microalgae on the immune system of aquatic organisms can also be designed using these data.

## 1 Data Description

All raw data of the figures and tables are provided in the Mendeley datasets. This data is showing the specific growth rate (SGR) and survival rate of Nile Tilapia (*Oreochromis niloticus*) fry cultured in different treatments where CF, T25, T50, N25 and N50 of this data is representing five treatments of control feed (CF) with no replacement of microalgae, 25% replacement of *Tetraselmis* sp., 50% replacement of *Tetraselmis* sp.*,* 25% replacement of *Nannochloropsis* sp. and 50% replacement of *Nannochloropsis* sp., respectively.

**Fig. 1:**
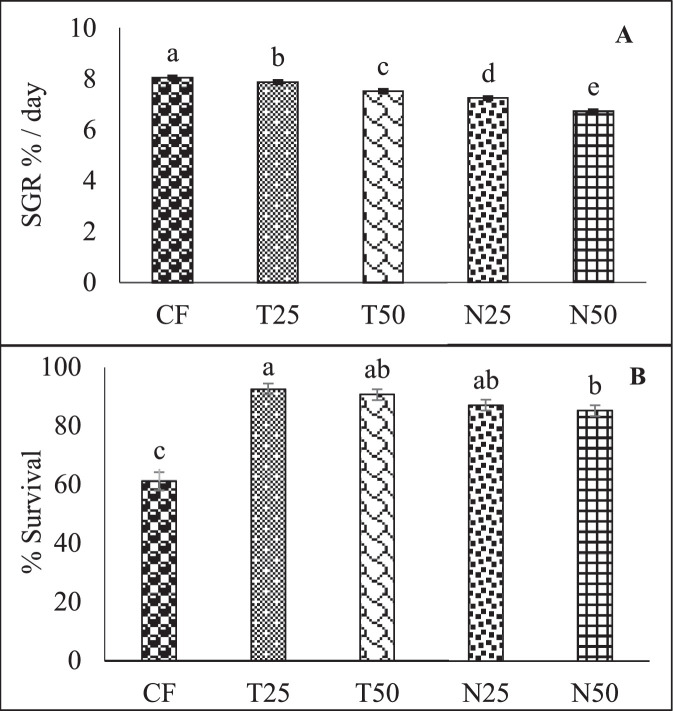
Specific growth rate (A) and survival rate (B) of Nile Tilapia (*Oreochromis niloticus*) fry fed with different dietary level of *Tetraselmis* sp. and *Nannochloropsis* sp. Values are mean with error bar (SE = σ/√n). Different letters within each series indicate significant (p < 0.05) difference.

Fig. 1 A and B described the percentage of specific growth rate and survival rate of Nile Tilapia (*Oreochromis niloticus*) fry cultured in different treatments. Data were presented as mean with error bar (SE = σ/√n) where the values varied significantly (p < 0.05) among the five treatments.

Physical and chemical parameters such as temperature (°C), dissolve oxygen (DO) (mg/L), pH, total ammonia nitrogen (TAN) (mg/L), nitrite nitrogen (NO2-N) (mg/L) and soluble reactive phosphorus (SRP) (mg/L) are shown in Table 1 for the different treatments during the experimental period. Data on the physical parameters such as temperature, dissolved oxygen and pH did not differ significantly (p > 0.05) during the experimental period among the treatments. However, the data of chemical parameters such as TAN, NO_2_–N and SRP showed significant (p < 0.05) difference among all the treatments.

**Table 1:** Physico-chemical parameters of Nile Tilapia (*Oreochromis niloticus*) fry cultured in different treatments for 56-day experimental duration. Values are means ± standard error. Different letters used in each row indicate the significant (p < 0.05) difference.

	Treatment				
Parameters	CF	N25	N50	T25	T50
Temperature (°C)	27.64±0.07^a^	27.65±0.09^a^	27.65±0.09^a^	27.80±0.09^a^	27.81±0.13^a^
DO (mg/L)	6.60±0.03 ^a^	6.58±0.06 ^a^	6.55±0.05 ^a^	6.55±0.03 ^a^	6.55±0.04 ^a^
pH	8.54±0.05^a^	8.50±0.05^a^	8.44±0.07^a^	8.42±0.05^a^	8.42±0.07^a^
TAN (mg/L)	0.66±0.01^a^	0.54±0.00^c^	0.59±0.00^b^	0.46±0.00^d^	0.54±0.00^c^
NO_2_-N (mg/L)	0.51±0.01^a^	0.44 ± 0.00^c^	0.47±0.00^b^	0.42±0.00^d^	0.42±0.01^cd^
SRP (mg/L)	0.15±0.00^a^	0.10±0.00^d^	0.13±0.00^b^	0.10±0.00^d^	0.11±0.00^c^

**Fig. 2:**
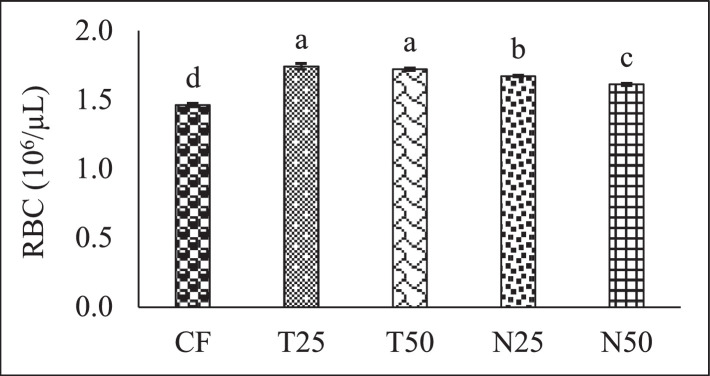
Red blood cell (RBC) of Nile Tilapia (*Oreochromis niloticus*) fry fed different dietary level of microalgae *Tetraselmis* sp. and *Nannochloropsis* sp*.* throughout the experimental duration. Values are mean with error bar (SE = σ/√n). Different letters within each series indicate significant (p < 0.05) difference.

**Fig. 3:**
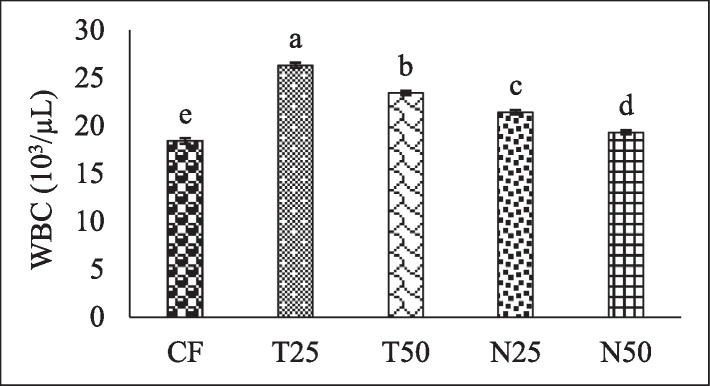
White blood cell (WBC) of Nile Tilapia (*Oreochromis niloticus*) fry fed different dietary level of microalgae *Tetraselmis* sp. and *Nannochloropsis* sp. throughout the experimental duration. Values are mean with error bar (SE = σ/√n). Different letters within each series indicate significant (p < 0.05) difference.

**Fig. 4:**
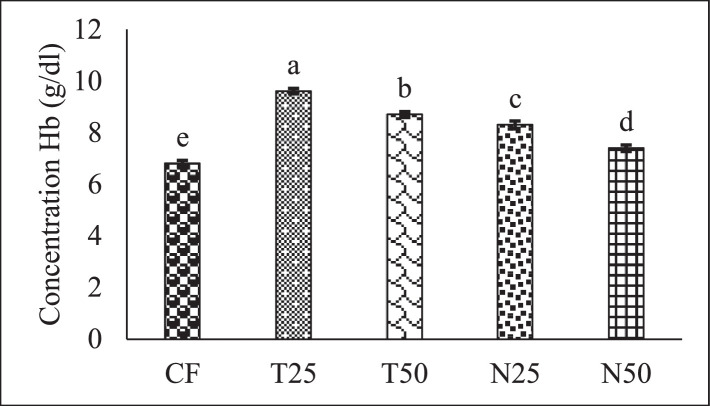
Hemoglobin (Hb) concentration of Nile Tilapia (*Oreochromis niloticus*) fry fed different dietary level of microalgae *Tetraselmis* sp. and *Nannochloropsis* sp. throughout the experimental duration. Values are mean with error bar (SE = σ/√n). Different letters within each series indicate significant (p < 0.05) difference.

**Fig. 5:**
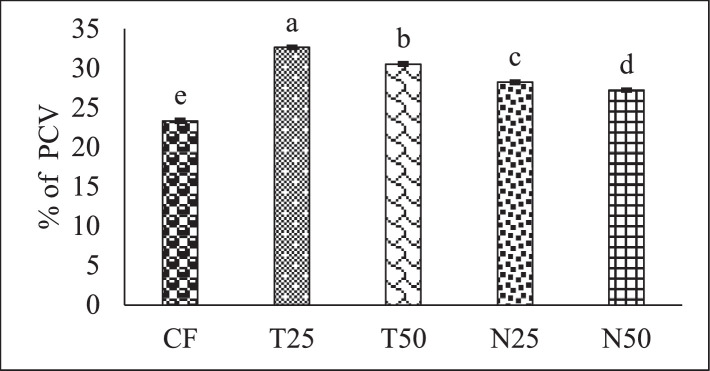
Percentage of packed cell volume (PCV) of Nile Tilapia (*Oreochromis niloticus*) fry fed different dietary level of microalgae *Tetraselmis* sp. and *Nannochloropsis* sp*.* throughout the experimental duration. Values are means with error bar (SE = σ/√n). Different letters within each series indicate significant (p < 0.05) difference.

**Fig. 6:**
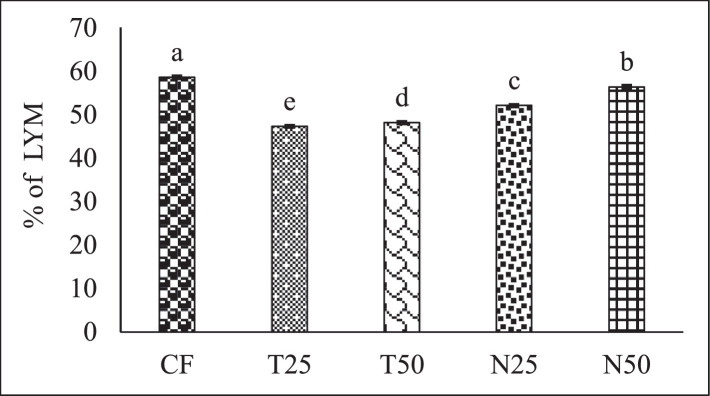
Percentage of lymphocytes (LYM) of Nile Tilapia (*Oreochromis niloticus*) fry fed different dietary level of microalgae *Tetraselmis* sp. and *Nannochloropsis* sp. throughout the experimental duration. Values are means with error bar (SE = σ/√n). Different letters within each series indicate significant (p < 0.05) difference.

**Fig. 7:**
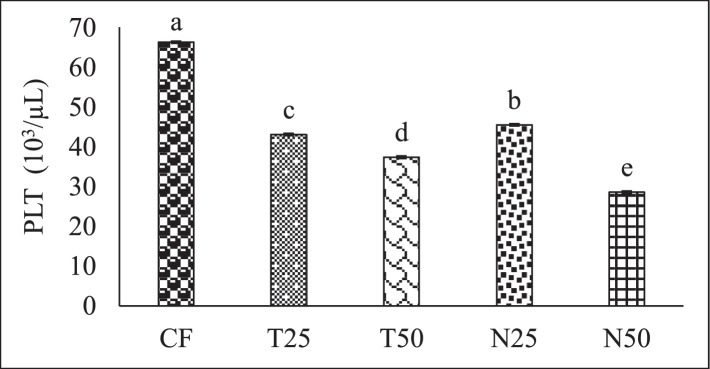
Platelet (PLT) of Nile Tilapia (*Oreochromis niloticus*) fry fed different dietary level of microalgae *Tetraselmis* sp. and *Nannochloropsis* sp. throughout the experimental duration. Values are means with error bar (SE = σ/√n). Different letters within each series indicate significant (p < 0.05) difference.

Figs. 2, 3, and 4 showed the red blood cell (10^6^/µL), white blood cell (10^3^/µL) and hemoglobin (g/dl) level of Nile Tilapia (*Oreochromis niloticus*) fry fed with different percentage (0, 25 and 50) of *Tetraselmis* sp. and *Nannochloropsis* sp. respectively. Figs 5, Fig. 6, Fig. 7 described the percentage of packed cell volume, lymphocytes and level of platelet (10^3^/µL) of Nile Tilapia (*Oreochromis niloticus*) fry fed with different percentage (0, 25 and 50) of *Tetraselmis* sp. and *Nannochloropsis* sp. respectively. Data are presented as mean with error bar (SE = σ/√n) and varied significantly from each other (p < 0.05) among the five treatments.

Finally, Table 2 is presenting different concentration of serum biochemical parameters, which are total protein (g/dl), albumin (g/dl), globulin (g/dl), albumin and globulin (A/G) ratio, blood glucose (mg/dl), triglyceride (mg/dl), cholesterol (mg/dl), urea (mg/dl), and blood urea nitrogen (mg/dl) level of Nile Tilapia (*Oreochromis niloticus*) fry.

**Table 2:** Effect of *Nannochloropsis* sp. and *Tetraselmis* sp. on serum biochemical indices of Nile Tilapia (*Oreochromis niloticus*) fry. Values are means ± standard error (SE = σ/√n). Different letters within each series indicate significant (p < 0.05) difference.

	Treatment				
Parameters	CF	N25	N50	T25	T50
Total protein (g/dl)	6.1±0.03^a^	4.8±0.01^d^	3.9±0.02^e^	5.6±0.04^b^	5.4±0.03^c^
Albumin (g/dl)	2.4±0.02^a^	2.1±0.03^c^	1.9±0.01^d^	2.±0.02^b^	2.3±0.02^b^
Globulin (g/dl)	3.6±0.02^a^	2.6±0.04^d^	2.0±0.02^e^	3.3±0.04^b^	3.1±0.04^c^
A/G Ratio	0.68±0.01^d^	0.79±0.02^b^	0.94±0.02^a^	0.69±0.01^cd^	0.74±0.01^c^
Blood glucose (mg/dl)	79.2±0.28^a^	72.8±0.08^c^	76.6±0.31^b^	67.5±0.08^e^	69.5±0.29^d^
Triglyceride (mg/dl)	213.6±0.27^a^	199.2±0.36^d^	194.1±0.27^e^	208.6±0.31^b^	201.9±0.43^c^
Cholesterol (mg/dl)	250.3±0.2^a^	208.2±0.24^d^	204.5±0.35^e^	230.5±0.29^b^	219.5±0.29^c^
Urea (mg/dl)	30.9±0.47^a^	24.6±0.44^cd^	23.8±0.18^d^	27.2±0.25^b^	25.3±0.18^c^
BUN (mg/dl)	14.4±0.26^a^	11.5±0.21^cd^	11.1±0.08^d^	12.7±0.12^b^	11.8±0.08^c^

## 2 Experimental Design, Materials and Methods

### 2.1 Source of microalgae and experimental location

*Tetraselmis* sp. and *Nannochloropsis* sp. were selected for the experiment and pure isolates were collected from laboratory of live feed research corner, Faculty of Fisheries, Chattogram Veterinary and Animal Sciences University. To prepare media for microalgae culture, seawater was collected from the nearest Sagorika Sea Beach, located at Kattoli in Chattogram. The collected sea water was taken into the lab and stored in plastic tanks overnight so that solid particles of seawater could settle down properly. At first, the seawater above from the settled solids was filtered using a filter bag. For fine filtration of seawater Whatman GMF Circles 4.7 cm paper was used in vacuum pump followed by autoclave (121°C 15 min). Filtered and sterilized seawater was used for microalgae culture. The feeding trial was conducted with proper fresh water circulation facilities from tap and stored in two plastic tanks for UV sterilization. After that, UV sterilized fresh water from tap was circulated into the fish rearing tank and whenever needed this process was continued for 56-day experimental duration at Wet Laboratory of the Faculty of Fisheries, Chattogram Veterinary and Animal Sciences University.

### 2.2 Conway media preparation

Conway medium was prepared by following proportions of Table 3 (modified from [1]). Microalgae culture media was prepared by adding main mineral nutrients (solution A) 1 mL, trace metal nutrients (solution B) 0.5 mL and vitamins (solution C) 0.1 mL in 1 L filtered and sterilized seawater.

**Table 3:** Chemical composition of Conway medium.

Solution A-Macronutrients	
Compound name and molecular formula	Proportions
Sodium/Potassium nitrate (NaNOз/KNOз)	100.00 g/116.00 g
EDTA Disodium salt (C_10_ H_16_N_2_O_8_)	45.00 g
Boric acid (H_3_BO_3_)	33.60 g
Sodium di-hydrogen orthophosphate (NaH_2_PO_4_.4H_2_O)	20.00 g
Ferric chloride hexahydrate (FeCL_3_.6H_2_O)	1.30 g
Manganese (II) chloride tetrahydrate (MnCL_2_.4H_2_O)	0.36 g
Deionized/distilled water	1 L

Solution B-Trace metal solution	

Compound name and molecular formula	Proportions

Zinc chloride (ZnCl_2_)	2.10 g
Cobalt (II) chloride hexahydrate (CoCl_3_.6H_2_O)	2.00 g
Ammonium molybdate tetrahydrate ((NH_4_)_6_MO_7_O_24_.4H_2_O)	0.90 g
Copper (II) sulfate pentahydrate (CuSO_4_.5H_2_O)	2.00 g
Deionized/distilled water	1L

Solution C-Vitamin's solution	

Compound name and molecular formula	Proportions

Thiamine, Vitamin B1	200 mg
Cyanocobalamin, Vitamin B12	10 mg
Deionized/distilled water	100 mL

### 2.3 Mass culture of microalgae

Mass culture of *Tetraselmis* sp. and *Nannochloropsis* sp. was done in large scale in 20 L clear plastic jar using Conway medium [2]. The culture was gradually scaled up from an initial starter culture volume of 20 mL to 20 L. Initially, 20 mL of microalgal stock culture was mixed with 30 mL medium in each flask (total culture volume 50 ml), with batch cultures of increasing volume; 250 ml, 500 mL, 1 L followed by bigger container of 20 L. During exponential period of development, cultures were transferred into the next batch. The microalgae were harvested by centrifugation [3] at stationary phase. Harvested biomass was then oven dried using a hot air oven (Natural Convention Oven LNO-150) at 60°C temperature for overnight, [4] and later, stored at normal refrigerator (4°C) for further use.

### 2.4 Formulation of diets

In order to include varying concentrations of microalgae *Nannochloropsis* sp. and *Tetraselmis* sp. five experimental diets have been formulated, replacing fishmeal with 25 and 50%, although there was no replacement in control feed (CF) (Table 4). The oven-dried microalgae biomass was grinded into fine particles (diameter of 0.4–0.5 mm) using a mortar and pestle, and then stored at normal refrigerator (4°C) until further use for feed preparation. Proximate composition of the test diets (% dry matter basis) for Nile Tilapia (*Oreochromis niloticus*) fry was analyzed and shown in Table 4.

**Table 4:** Feed formulation and proximate composition of the test diets (% dry matter basis) for Nile Tilapia (*Oreochromis niloticus*) fry.

	Proportions				
Ingredients	CF	N25	N50	T25	T50
Commercial fish meal	67.55	50.67	33.78	50.67	33.78
*Nannochloropsis* sp.	_	16.88	33.77	_	_
*Tetraselmis* sp.	_	_	_	16.88	33.77
Wheat flour	9.65	9.65	9.65	9.65	9.65
Corn flour	9.65	9.65	9.65	9.65	9.65
Rice bran	9.65	9.65	9.65	9.65	9.65
Dicalcium phosphate	1	1	1	1	1
Vitamin and mineral premix	2	2	2	2	2
Molasses	0.5	0.5	0.5	0.5	0.5
Total	100	100	100	100	100
Proximate composition (%)					
Protein	40.00	36.03	33.45	38.00	36.90
Lipid	11.21	14.37	16.22	12.29	13.11
Carbohydrate	20.43	22.33	23.18	23.19	26.22

### 2.5 Proximate composition analysis of formulated diets

#### 2.5.1 Protein analysis

Protein analysis was done according to Lowry et al. [5]. Briefly, 5,6 mg of oven-dried microalgae sample was taken for each sample analysis and 25 ml solution was prepared by mixing with deionized water. 0.5 ml of aliquot from each sample was taken from the prepared 25 ml sample for protein analysis. Reactive 1 (1% potassium sodium tartarate) and 2 (2 g sodium carbonate per 100 ml 0.1 N NaOH) were previously made. By adding 1 ml of Reactive 1–50 ml Reactive 2 mixed reagent preparation was done. Then, 0.5 ml of sample was added with 0.5 ml of 1 N sodium hydroxide and it was kept at 100°C in a water bath for 5 min. It was then cooled in a water bath and 2.5 ml of the prepared mixed reagent was added within 10 min after cooling. The mixed solution was added with 0.5 ml of Folin reagent and was kept in dark place for 30 min. The absorbance of the mixed solution was taken with spectrophotometer (T80 UV/VIS Spectrophotometer) at the wavelength of 750 nm.

#### 2.5.2 Lipid analysis

According to Bligh and Dyer [6] and Folch et al. [7] lipid content was analyzed. Briefly, aluminum dishes were prepared and labeled for each sample. Initial weight of each labeled dishes was recorded. In a centrifuge tube pre weighted 50 mg sample was taken and using deionized water diluted 5 times of the volume. Tissue homogenizer was used to mix 3 ml 1:2 chloroform: methanol (v/v) with the sample consistently. Then centrifuged for 4 min at 1000 rpm at 4°C temperature. Using a Pasteur pipette supernatants were shifted into clean centrifuge tube and kept into the ice. Repeatedly 3 ml 2:1 methanol: chloroform (v/v) were mixed with the sample consistently. The tubes were again centrifuged and the supernatants were moved to the previous supernatant tubes. 1.5 ml of 0.9% NaCl was mixed with a vortex mixture (VM-10) in this combined supernatant. The tubes were then kept in the refrigerator at 4°C for an hour. Tubes were centrifuged after an hour at a temperature of 4°C at 1000 rpm for 10 min. Methanol and chloroform were removed from the upper layer, while aluminum dishes had been used to transport the low layer. A hot air oven was used to evaporate the solvent at 40°C. The aluminum plate was then weighted to obtain the final weight. At the end, the initial weight was deducted from the final weight to obtain the lipid weight of the samples.

#### 2.5.3 Carbohydrate analysis

Based on the method of Dubois et al. [8] carbohydrate analysis was conducted. For each analysis, 5-6 mg of oven-dried microalgae sample was taken and 25 ml solution was prepared by mixing with deionized water. 5% phenol solution and concentrated sulphuric acid was prepared previously of the analysis. One milliliter aliquot was taken into the test tube from the prepared sample. One milliliter of 5% phenolic solution and 5 ml of concentrated sulphuric acid was added to the test tube and kept into the ice bath for cooling. After cooling the optical density was measured at 488 nm wavelength using spectrophotometer (T80 UV/VIS Spectrophotometer).

### 2.6 Source of fish and experimental design

For this experiment fish were collected from Niribili Tilapia Hatchery, Cox's bazar. Nile tilapia (*Oreochromis niloticus*) fry (14 days old) were acclimatized for 2 days in a storage plastic tank before stocking. During conditioning, continuous O_2_ supply was provided in the tank using aerator pump connected with air stone. Fish were fed commercial feed twice daily during conditioning period. After the end of conditioning period fries were starved overnight. After that, average individual weight (0.023±0.0001 g) of 270 fry was recorded and randomly distributed into 15 rectangular glass aquaria with a culture volume of 18 L and the stocking density was one fish per liter. However, the total water holding capacity of the culture tank was 30 L (45 × 30 × 30 cm). Eighteen fry per tank in triplicate were equally stocked in five different treatments such as only commercial feed (CF), T25 (75% CF + 25% *Tetraselmis* sp. biomass), T50 (50% CF + 50% *Tetraselmis* sp. biomass), N25 (75% CF + 25% *Nannochloropsis* sp. biomass), N50 (50% CF + 50% *Nannochloropsis* sp. biomass).

#### 2.6.1 Feed and feeding

Five dietary concentration of microalgae were administered which were, N25 with 25% *Nannochloropsis* sp., N50 with 50% *Nannochloropsis* sp., T25 with 25% *Tetraselmis* sp., T50 with 50% *Tetraselmis* sp. and CF without any microalgae-based treatment. Every 10 days interval feed was prepared. Fish were fed 4 times in a day (at 8 AM, 11 AM, 2 PM and 5 PM). Feeding rate was 15% based on the total fish body weight. Total feed for a day was divided into four parts and fed the fish 4 times a day. Mashed feed was directly applied to the experimental aquarium. The left-over feed particles, feces and debris were removed by siphoning method daily. One third of total culture water was exchanged constantly with fresh UV sterilized tap water and continuous diffusion of oxygen from air to water through electrical aerator pump provided sufficient oxygen in the experimental unit. Thus, water quality of the culture tank was maintained.

### 2.7 Physical and chemical analyses

Temperature, salinity, pH, and dissolved oxygen in the culture tanks were measured daily using multi parameter. Total ammonia nitrogen, NO_2_–N and SRP were analyzed once in a week following the method of Parsons et al. [9].

### 2.8 Biological analyses

The specific growth rate (SGR) of the tilapia fry was calculated based on dry weight according to Ricker [10]. Tilapia fry survival was calculated at the end of the experiment. Nile Tilapia fry body weight (g) was performed at the beginning and end of the experiment by using electronic balance. Survival rate was measured by counting the live fish numbers at the beginning and end of the experiment for 56-day experimental duration. Following formula were used to measure the specific growth rate and survival rate values:

(a) $\text{SGR}\left(\%/\text{day}\right)=\frac{\left(\text{Ln}\left(w_{t}\right)-\text{Ln}\left(w_{i}\right)\right)}{t}\times 100$

Where, Ln(w_t_) is the natural logarithm of the final weight at time t and Ln(w_i_) is the natural logarithm of the initial weight. t is the time in days.

(a) $\text{Survival}\;\text{rate}\left(\%\right)=\frac{\text{Number}\;\text{of}\;\text{fish}\;\text{at}\;\text{the}\;\text{end}\;\text{of}\;\text{the}\;\text{experiment}}{\text{Number}\;\text{of}\;\text{fish}\;\text{at}\;\text{the}\;\text{beginning}\;\text{of}\;\text{the}\;\text{experiment}}\times 100$

### 2.9 Blood collection and analysis

Fish blood sample was collected at the end of the experimental period of 56-days. Five fish/tank was chosen randomly and anaesthetized by using clove oil (eugenol solution). Blood was obtained from the caudal vein of the fish by using 1 ml sterile syringe. Collected blood was immediately transferred into 2 ml EDTA tube (EDTA K3 POVEN vacuum tube) and also in EDTA-free clot activator (CURE) tubes for hematology and serum biochemical parameter analysis respectively. Blood collected in EDTA tubes were stored in refrigerator at 4°C until analysis. EDTA blood samples were centrifuged at 2000 × g for 10 min at 4°C [11]. Then blood plasma was collected by using micropipette and stored in refrigerator at -20°C in 1.5 ml Eppendorf tube until analysis. Hematological parameters: Red blood cell (RBC), Hemoglobin (Hb), Packed Cell Volume (PCV), White Blood Cell (WBC), Lymphocyte (LYM) were counted/measured using Hematology analyzer (NIHON KOHDEN). Serum biochemical parameters such as: total serum protein, albumin, globulin, triglyceride, cholesterol, urea, blood glucose, blood urea nitrogen of fish was determined using a biochemical analyzer (Humalyzer 3000).

### 2.10 Statistical analysis

In order to calculate mean and standard error of the mean of data MS excel was used. The normality of the data was verified through the Kolmogorov-Smirnov test. The Levene test checked the homogeneity of variation. Then the one-way ANOVA test was introduced to verify the significant differences of growth rate, survivability and hemato-biochemical indices at the level of likelihood p < 0.05 and to compare the means, Duncan multiple range test was done. For statistical tests IBM SPSS (v. 26.0) software was used.

## Ethical Statement

These data were collected complying ARRIVE guidelines carried out in accordance with the U.K. Animals (Scientific Procedures) Act, 1986 and associated guidelines, EU Directive 2010/63/EU for animal experiments, or the National Institutes of Health guide for the care and use of Laboratory animals (NIH Publications No. 8023, revised 1978).

## CRediT authorship contribution statement

**Kafia Islam Amira:** Methodology, Data curation, Writing – original draft. **Mohammad Redwanur Rahman:** Conceptualization, Supervision, Validation, Writing – review & editing. **Suchandan Sikder:** Conceptualization, Supervision, Validation, Writing – review & editing. **Helena Khatoon:** Supervision, Writing – review & editing. **Jinat Afruj:** Data curation. **Mohammad Ekramul Haque:** Data curation. **Tashrif Mahmud Minhaz:** Validation, Writing – review & editing.

## Declaration of Competing Interest

The authors disclose that they have no known conflict of interests that may have influenced either the data collection or the presentation of the data.
